# Preparation and characterization of functionalized heparin-loaded poly-Ɛ-caprolactone fibrous mats to prevent infection with human papillomaviruses

**DOI:** 10.1371/journal.pone.0199925

**Published:** 2018-07-02

**Authors:** Daniela Gonzalez, Jorge Ragusa, Peter C. Angeletti, Gustavo Larsen

**Affiliations:** 1 Department of Chemical and Biomolecular Engineering, University of Nebraska-Lincoln, Lincoln, Nebraska, United States of America; 2 Nebraska Center for Virology, School of Biological Sciences, University of Nebraska-Lincoln, Lincoln, Nebraska, United States of America; Wayne State University, UNITED STATES

## Abstract

In this study, heparin-loaded poly-ɛ-caprolactone (PCL) fibrous mats were prepared and characterized based on their physical, cytotoxic, thermal, and biological properties. The main objective of the work described here was to test the hypothesis that incorporation of heparin into a PCL carrier could serve as bio-compatible material capable of inhibiting Human Papillomavirus (HPV) infection. The idea of firmly anchoring heparin to capture soluble virus, vs. a slow heparin release to inhibit a virus in solution was tested. Thus, one material was produced via conventional heparin matrix encapsulation and electrohydrodynamic fiber processing in one step. A second type of material was obtained via heparin crosslinking. This was achieved by running a carbodiimide/N-hydroxysuccinimide (EDC/NHS) coupling reaction on preformed PCL fibers. *In vitro* HPV16 L1 protein binding capacity studies were performed. Infectivity assays were done using HPV16 pseudoviruses (PsVs) carrying a GFP plasmid to directly test the ability of the fibrous mats to prevent internalization of HPV PsVs. The crosslinked heparin material presented a dissociation constant (K_d_) value comparable to those found in the literature for different heparin-protein L1 peptide interactions. Both materials significantly reduced internalization of HPV PsVs, with a reduction of 94% of PsVs internalization when matrix encapsulated heparin-loaded material was present. Differences in performance between the two proposed structures are discussed.

## Introduction

Human Papillomavirus (HPV), the most common worldwide sexually transmitted virus, infects the squamous mucosal layers of the stratified epithelium. Of the more than 200 HPV genotypes known, about 16 are associated with cancers of the penis, vulva, anus, vagina, cervix, and oropharynx [1–3]. About 14 million new HPV infections occur annually in the United States (US) [4]. Most HPV infections are asymptomatic and typically clear without any treatment. However, persistent infection with high-risk (HR) HPVs is a key factor for the development of pre-cancers and cancers. Infections with HR-HPV types are the primary cause of cervical cancer, the second most common cancer and the leading cause of cancer deaths in women in developing countries. In 2015, there were 526,000 cervical cancer cases world-wide and 239,000 attributable deaths, with 85% of deaths occurring in developing countries [5,6]. According to the American Cancer Society, in 2017, more than 12,800 women in the US were expected to be diagnosed with cervical cancer and an estimated of 4,210 were expected to die from it [7]. Current approaches to reduce the incidence of cervical cancer are based on cervical cancer screening methods (Papanicolaou and HPV tests) and prophylactic HPV vaccines [8–11]. Gardasil^®^ and Cervarix^®^ are the two vaccines approved for the prevention of the most common HPV types that cause cervical cancer. However, both vaccines are not therapeutically effective, nor do they cross-protect against all HPV genotypes. No vaccine directly treats cervical cancer itself. Thus, a remaining challenge is to develop effective tools for both prevention and intervention against existing HPV infections.

The icosahedral HPV particles are composed of structural proteins, L1 (major) and L2 (minor), which encapsidate the circular double-stranded DNA genome. Papillomaviruses infect the basal epithelia of the stratified epithelium, and are tightly linked to differentiation of keratinocytes for completion of the viral life cycle. The initial attachment of HPV is promoted through interactions between capsid proteins L1 and sulfated sugars on the cell surface [12].

Heparin is a linear, polydispersed polysaccharide which consists of a repeating disaccharide unit with variable amounts of sulfate substituent groups. Besides its anticoagulant properties, it has the ability to interact with several proteins to impact a wide range of biological molecules, including enzymes, extracellular matrix proteins, growth factors, and surface proteins of many pathogens. Heparan sulfate (HS), a molecular variant of heparin, is commonly found on cell surfaces, and has been shown to serve as the primary attachment molecule for HPV infection [13–15]. Both heparin and HS have been shown to inhibit HPV infection in keratinocytes [16,17]. Heparin as a barrier against HPV constitutes an excellent target molecule, since it has an affinity for HPV capsids with dissociation constants (K_d_) estimated to be as low as 10^−9^ [18,19].

Electrohydrodynamics (EHD) is the most common, inexpensive, simple, cost-effective way to scale-up production of fibrous mats. The technique is well established, and has been reviewed elsewhere [20–23]. Our group has exploited EHD to produce a variety of functional structures for the past fifteen years [24–28]. One of the main advantages of EHD-processed fibers is that they have a high surface area-to-volume ratio, which enhances their interaction with different additives relevant to their intended application. This technique also offers an opportunity to incorporate important biomolecules into the fibers. PCL is a hydrophobic, semi-crystalline linear aliphatic polyester which has been widely used in the biomedical field. Some important properties of this polymer are its good biocompatibility and structural stability. PCL degrades at a slower rate than most other well-known resorbable polymers, making it attractive for applications in which preservation of the device’s structural integrity is important [29,30]. Heparin-loaded fibers have been produced by other groups [31–34], but they have not been evaluated for use in suppression of HPV infection, which is the primary objective of our work. The high surface-to-volume ratio is an important characteristic which was selected during mat fabrication for the current work. In our application, the very high aspect ratio (length/diameter) of EHD-processed fibers offer a higher surface area through which HPVs may interact tightly with the heparin component. In addition, the simplicity of the EHD method avoids the use of harsh chemical reagents, which would have a detrimental effect on functional assays.

A material to inhibit HPV infection can adopt two basic configurations: A) As a typical controlled release device, where heparin, the virus-inhibiting molecule, is liberated in the virus-containing environment, or B) as a “capture” mesh, where the heparin is firmly anchored onto the surface of the material. Either device type could *a priori* serve its intended purpose, albeit via entirely different mechanisms. Thus, the objective of this paper is to explore both possibilities, in an effort to determine the best possible alternative for HPV inhibition. Two different approaches to produce heparin-loaded poly-ε-caprolactone (PCL) fibrous materials were applied: one involved a standard matrix encapsulation of heparin within the fiber structure, while the second was based on the use of chemical cross-linking of heparin to the PCL backbone. Both materials were characterized on the basis of their physical, cytotoxic, thermal and biological properties. The ability of the two materials to bind to HPV L1 capsid proteins and to prevent HPV infection was also evaluated.

## Materials and methods

### 2.1 Materials

Poly-ε-caprolactone (PCL, Mn 80000 GPC), heparin sodium salt from porcine intestinal mucosa, methanol (MeOH, Fischer Scientific), dichloromethane anhydrous (DCM, ≥99.8%), sodium heparin from intestinal mucosa bound to Fluorescein Isothiocyanate (HepF, MW 3–30 kDa, 179–181 U/mg, Polysciences) and the chemicals 2-(N-Morpholino) ethanesulfonic acid (MES), N-(3-Dimethylaminopropyl)-N-ethylcarbodiimide hydrochloride (EDC), N-hydroxysuccinimide (NHS) and toluidine blue O (technical grade) were purchased from Sigma-Aldrich (St. Louis, MO) unless stated.

### 2.2 Fibrous mat preparation

#### 2.2.1 PCL and PCL-heparin polymeric membranes

PCL solutions at a concentration of 8.8% (w/v) in DCM:MeOH (3:1) solvent mixture were prepared. For the incorporation of heparin, a method previously described was adapted to our needs [32]. PCL heparin solutions at a concentration of 7% (w/v) PCL with heparin at 1 wt% (relative to dry PCL) in DCM:MeOH (3:1) solvent were prepared. Heparin was first dissolved in deionized (DI) water (2% w/v), and then mixed with aliquots of MeOH and DCM:MeOH (3:1) solvent mixture. The volume ratio of water:MeOH:(DCM:MeOH) was 1:3:22. This final solution was used to dissolve the PCL polymer. All solutions were freshly prepared and magnetically stirred until complete homogenization. All solutions were EHD-processed and collected over the surface of a circular, aluminum foil wrapped, grounded collector electrode (copper disk, OD = 15cm). The flow rate (1mL/hr) was controlled using a syringe pump (Cole-Parmer 74900–00, Vernon Hills, IL). The working distance needle tip-to-collector was 15 cm, and the applied voltage was 12kV (Gamma High Voltage Research, ES30P-5W/PRG, Ormond Beach, FL). The materials were denominated as PCL and PCL-Hep mats. For cell culture experiments PCL and PCL-Hep mats were collected and cut in a biosafety hood. All mats were collected for 6 hrs at room temperature and stored in sealed zip-lock bags at 4°C until further use.

#### 2.2.2 PCL crosslinked membranes

To fabricate the heparin crosslinked material (PCL-Hep-CL), PCL-Hep mats were crosslinked by applying a well-known method via the EDC/NHS coupling reaction (S1 Fig and S1 Text) [33,35,36]. Before the crosslinking reaction, circular PCL-Hep mats (OD = 1cm) were saturated with 0.05 M MES buffer (pH 5.5) for 30 min and then incubated at room temperature with gentle shaking, in freshly prepared solution of heparin (1% w/v), 0.5 M EDC and 0.5 M NHS in MES buffer for 20 hrs. Discs were washed (x3) with DI water and placed at -21°C. Samples were freeze-dried overnight and stored in the refrigerator.

### 2.3 Materials characterization

#### 2.3.1 Fiber size and morphology

Fibers diameter and morphology of all mats were assessed by scanning electron microscopy (SEM) images taken with Quanta 200 FEG Environmental Scanning Electron Microscope using an accelerating voltage of 15–20 kV and working distance of approximately 10 mm. A Cressington 108 Auto Sputter Coater was used for sample coating with metallic gold for 30 seconds before SEM characterization. Statistical analysis of fiber diameter measurements from SEM images was performed on sets of at least 200 counts within each specimen.

#### 2.3.2 Fourier Transform Infrared spectroscopy

Surface chemical analysis of PCL, PCL-HEP-CL and heparin powder was performed by Attenuated Total Reflection-Fourier Transform Infrared (ATR-FTIR) Spectroscopy using a Nicolet iS50 FT-IR Spectrometer (Thermo Scientific, Waltham, MA). ATR-FTIR transmittance spectra were collected with the built-in diamond ATR crystal module using 16 scans and 4 cm^-1^ resolution over the range of 4000 and 500 cm^-1^ wave number.

#### 2.3.3 Distribution and surface immobilization of heparin in PCL mats

To assess the distribution of heparin in PCL fibers, HepF conjugate was used instead of regular heparin. PCL-HepF mats, loaded with the fluorescent conjugate of heparin, were made following the same procedure as the one used for PCL-Hep mats. PCL-HepF fibers were collected in a microscope glass slide and analyzed using confocal microscopy (Olympus IX 81). The amount of immobilized heparin was determined using toluidine blue (TB) assay [37–39]. PCL-Hep and PCL-Hep-CL circular discs (OD = 1cm) were incubated with 5 mL of freshly prepared solution of 0.005 wt% TB in aqueous 0.01 N HCl/0.2 wt% NaCl for 4 hrs. The membranes were rinsed (x3) with DI water and incubated in 5 mL of a 1:4 (v/v) mixture of aqueous 0.1 N NaOH and ethanol solution, until complete mat discoloration. The absorbance of the resultant solution was measured at 530 nm, and the amount of heparin was calculated using a calibration curve obtained using the same procedure, with solutions of untreated heparin of known concentrations.

#### 2.3.4 Contact angle measurement

To evaluate the hydrophilicity of the fibrous mats, contact angles of PCL, PCL-Hep and PCL-Hep-CL mats were measured using a simplified experimental set up [40]. Each measurement was performed by placing a 5 μL DI water droplet on the samples and measuring contact angles between the water droplets and mat surfaces.

#### 2.3.5 Thermal properties of PCL mats

Differential Scanning Calorimetry (DSC) runs were performed on Q100 machine (TA Instruments, New Castle, USA), calibrated for temperature and heat flow using indium (melting point 156.6°C, ΔHm 28.45 J/g). Two analytical runs were performed on each sample using an empty hermetic Al pan as reference. Two heating cycles were performed at a scanning rate of 10°C/min from 0 to 100°C under nitrogen gas flowing at 25 mL/min. DSC plots from the second heating cycle were exported using TA Universal Analysis 4.4v software. The melting temperature (T_m_) and melting enthalpy (ΔH_m_, area of melting peak) were determined from the respective thermograms. The degree of crystallinity (X_c_) was calculated as ΔH_m_/ΔH_mo_, where ΔH_mo_ is the enthalpy of melting of fully crystalline PCL, which is 139 J/g [41].

#### 2.3.6 Cytotoxicity assay of fibrous mats

Cell lines were purchased from ATCC (Manassas, VA). Mouse fibroblast cell line L-929 was cultured in Eagle's Minimum Essential Medium (EMEM, ATCC) supplemented with 10% fetal bovine serum (FBS, PAA Lab, Westborough, MA), 100IU units/mL penicillin, and 100 μg/mL streptomycin. Human cell line: 293 Human Embryonic Kidney cells expressing SV40 T-antigen (293FT) were cultured in Dulbecco’s modified Eagle’s medium (DMEM, Global Cell Solutions, Charlottesville, VA) supplemented with 10% FBS (DMEM-10), 100IU/mL penicillin, and 100μg/mL streptomycin. Both cell cultures were kept at 37°C and 5% CO_2_ in a humidified incubator. Cell viability was determined using a WST-8 assay kit performed as manufacturer’s instructions (Dojindo Molecular Technologies, Inc.). Briefly, mouse fibroblast and 293FT cells were seeded in 96-well plates at a density of 1 x 10^4^ cells/well. PCL, PCL-Hep and PCL-Hep-CL mat discs (OD = 0.5cm) were pre-incubated in DMEM for 24 hrs. Mat supernatants were added to cells and incubated for 3 days at 37°C and 5% CO_2_. Then 10 μL of WST-8 reagent was added to each well. Plates were incubated for 1 hr prior to OD450 measurement with an Elx800TM universal microplate reader (BioTek Instruments, Inc.). Cells viability was determined by comparing the absorbance of WST-8 reagent in the cell suspension with that of control wells (cells in media alone).

#### 2.3.7 Evaluation of heparin stability on fibrous mats

PCL-HepF mats (~20 mg) were immersed in 12 mL of phosphate buffered saline (PBS, pH = 7.4) and kept at 37°C over a period of 18 days (n = 3). Samples of 600 μL were extracted at specific time points, and replaced with fresh PBS. Fluorescence intensity of samples was measured with a fluorescence spectrophotometer (HITACHI, F-4500) at excitation and emission wavelengths of 490 and 515 nm, respectively. The HepF content was calculated using a calibration curve previously obtained. The release kinetics was calculated by regression analysis according to the Ritger and Peppas equation [42]: M_t_/M_∞_ = kt^n^, in which k is a constant related to the structure and geometrical characteristics of the system, n is the exponent, which depends on the release mechanism, and M_t_/M_∞_ is the fraction of the drug released at time t. The stability of heparin in PCL-Hep-CL mats was determined using TB assay. PCL-Hep-CL discs (OD = 1cm) were immersed in 6 mL of PBS (pH 7.4) at 37°C, over specific periods of time (n = 3). After each time’s interval, discs were taken out and rinsed with DI water. To determine the amount of heparin still present on the discs’ surfaces the same TB assay procedure used for heparin immobilization studies was followed.

### 2.4 Evaluation of heparin-loaded PCL materials with HPV16 virion proteins

#### 2.4.1 Glutathione S-transferase (GST)- L1 protein production

Bacterial, *Escherichia coli (E*. *coli*) BL21 (DE-3) cells were transformed with the pGEX-3T-16L1 vector to express GST moiety fused to the NH2-terminus of the HPV16 L1 protein. *E*. coli BL21 (DE-3) bacteria expressing the T7 phage RNA polymerase were used to achieve high levels of GST-16L1 fusion. Briefly, mid-log phase cultures of bacteria were incubated at 37°C with shaking. Cultures were induced to express GST-16L1 by addition of 0.5 mM Isopropyl β-D-1-thiogalactopyranoside (IPTG) for 2 hrs. Cells expressing exogenous proteins were prepared as lysates by extraction of the protein with 0.5% NP-40 in Tris-Buffered-Saline (TBS) solution. Lysates were clarified by centrifugation at 14,000Xg for 20 min at 4°C. The supernatant transferred to fresh centrifuge tubes which were stored at -80°C until further use. The L1 protein content in lysate was determined by comparison to bovine serum albumin (BSA) standards using Western Blot.

#### 2.4.2 Binding assay between heparin loaded materials and GST-L1 protein

PCL (control), PCL-Hep and PCL-Hep-CL mat discs (OD = 1cm) were preblocked with 1% non-fat milk in 1xTBS (pH 7.4) for 1 hr. Mats were washed and incubated with increasing concentrations of GST-L1 protein for 1 hr. To quantify the amount of bound protein, Western Blot was performed directly onto the mats. GST-L1 protein was detected using a rabbit polyclonal IgG anti-GST (1:1000 dilution, Santa Cruz Biotechnology, TX) as primary antibody, and an anti-rabbit IgG conjugated to horse-radish peroxidase (HRP) (1:2000 dilution, GE Healthcare Life Sciences, PA) as secondary antibody. Visualization using developing solution for chemiluminescence detection, according to the manufacturer’s protocol (Pierce), was done with a BIO RAD ChemiDoc ™ MP Imaging System. Antibody and L1 protein dilutions were made in TBS. The incubation steps were performed with rocking at 4°C. Experiments were run in triplicates, means and SE were calculated.

#### 2.4.3 HPV16 PsV production and purification

The methods adopted for the production of HPV16 PsVs are essentially based on protocols published elsewhere [43,44]. In brief, 293FT cell line was cultured in DMEM supplemented with 10% heat-inactivated FCS (HyClone), 1% nonessential amino acids (Invitrogen, CA), and 1% Glutamax-I (Invitrogen, CA). 250 μg/mL of hygromycin B (Roche, Indianapolis, IN) were added to promote maintenance of T antigen expression. Preplated 293FT cells were co-transfected (Lipofectamine 2000, Invitrogen, CA) with plasmid pShell encoding HPV16 L1/L2 capsid proteins genes and a reporter plasmid containing the green fluorescent protein (GFP). After 48 hrs, cells were resuspended and lysed in DPBS supplemented with 9.5mM MgCl_2_, 0.2% Brij 58, 0.2% Benzonase, and 0.1% Plasmid Safe exonuclease (Epicentre). For PsV maturation, cell lysate was incubated overnight at 37°C, then chilled, adjusted to 0.8M NaCl, and centrifuged at 2,000 x g for 15 min at 4°C. Clarified PsV supernatants were stored at -80°C until use.

#### 2.4.4 HPV16 PsV infection inhibition assay

PCL, PCL-Hep and PCL-Hep-CL mat discs (OD = 1cm) were preblocked with DMEM-10 in 96-well tissue culture plates at 37°C for 1 hr. A dilution of PsV stock in DMEM were pre-incubated with the different mats for 1 hr at 37°C and subsequently added to 293FT preplated cells, and incubated at 37°C, 5% CO_2_ for 48 hrs. Images of transduced cells under each condition were taken with a confocal microscope, and fluorescence intensities from the images were quantified with ImageJ 1.49v software.

### 2.5 Statistical analysis

Results are presented in the form of mean ± standard deviation (SD) or standard error (SE) with n-values listed below each corresponding figure. Statistical evaluation was performed using ANOVA test with p-values below 0.01 considered as significant.

## Results

### 3.1 Materials characterization

#### 3.1.1 Fiber diameters and morphology of the fibrous mats

**Fig 1:**
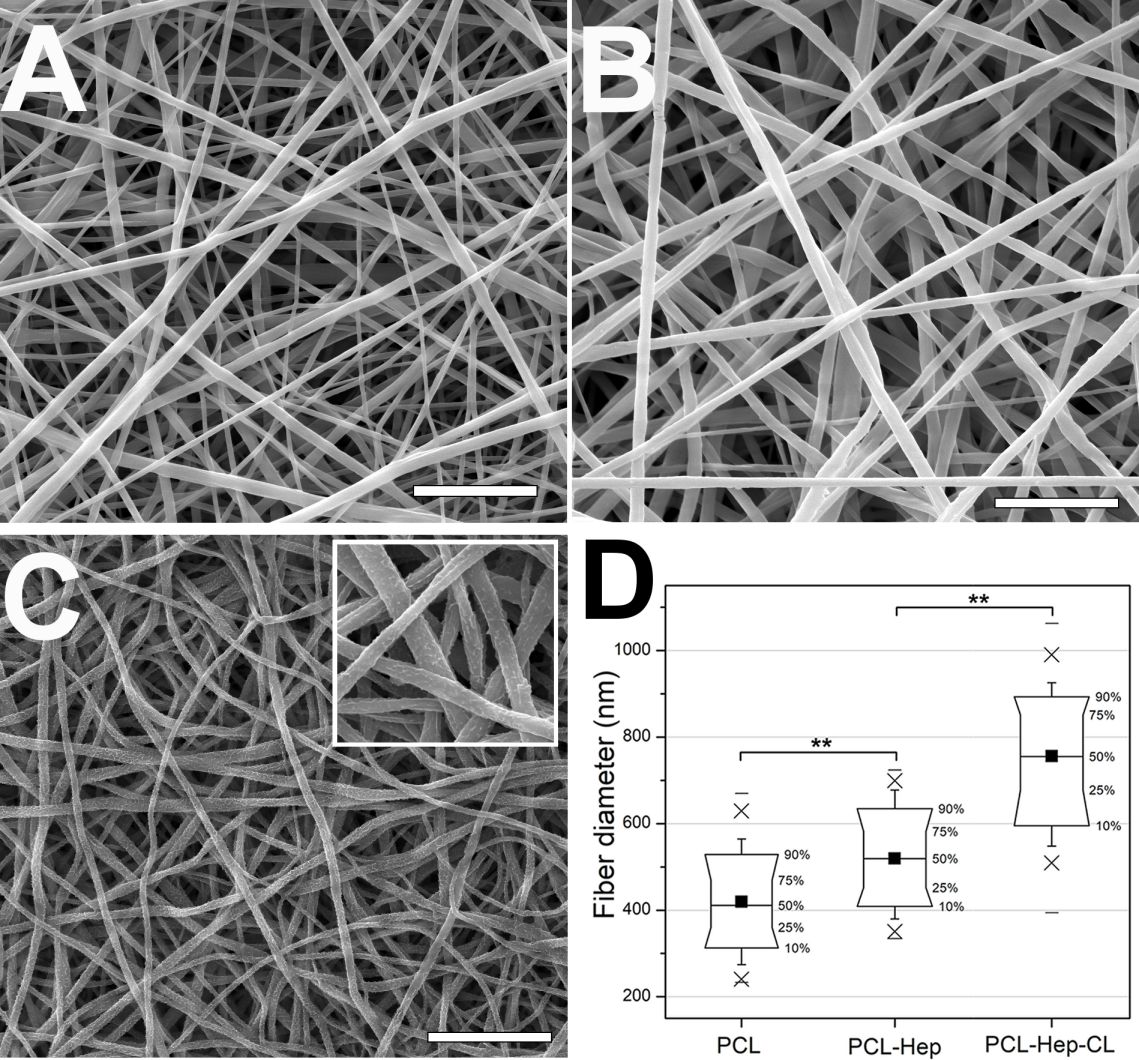
SEM images and fiber diameter distributions of PCL fibrous materials. (A) As-collected PCL mat, (B) as-collected PCL-Hep mat, (C) PCL-Hep-CL mat, inset: fibers with higher magnification. Scale bars represent 5μm (A and B) and 10 μm (C). (D) Fiber diameter distributions of mats, PCL: 419.40 ± 86.64nm, PCL-Hep: 519.64 ± 85.07nm, and PCL-Hep-CL: 756.03 ± 117.76nm. Values represent means ± SD. Average fiber diameter (■), box range: 10–90%, whisker range: 5–95%, (x) 1–99%, (−) minimum and maximum values of fiber diameter within investigated population. There were significant differences between the different types of mats (**, p<0.01).

SEM images of PCL, PCL-Hep and PCL-Hep-CL mats are shown in Fig 1. The morphology of the as-collected PCL and PCL-Hep fibers (Fig 1A and 1B) was characterized by a continuous and smooth texture. However, the crosslinked fibers displayed noticeable sub-micron roughness (Fig 1C and inset). The average fiber diameter in PCL mats was 419.40 ± 86.64 nm (Fig 1D). While the average fiber diameters in PCL-Hep and PCL-Hep-CL mats were 519.64 ± 85.07nm and 756.03 ± 117.76nm, respectively (Fig 1D). PCL and PCL-Hep mats presented a narrower fiber diameter distribution with respect to the crosslinked mat.

#### 3.1.2 FTIR spectra

**Fig 2:**
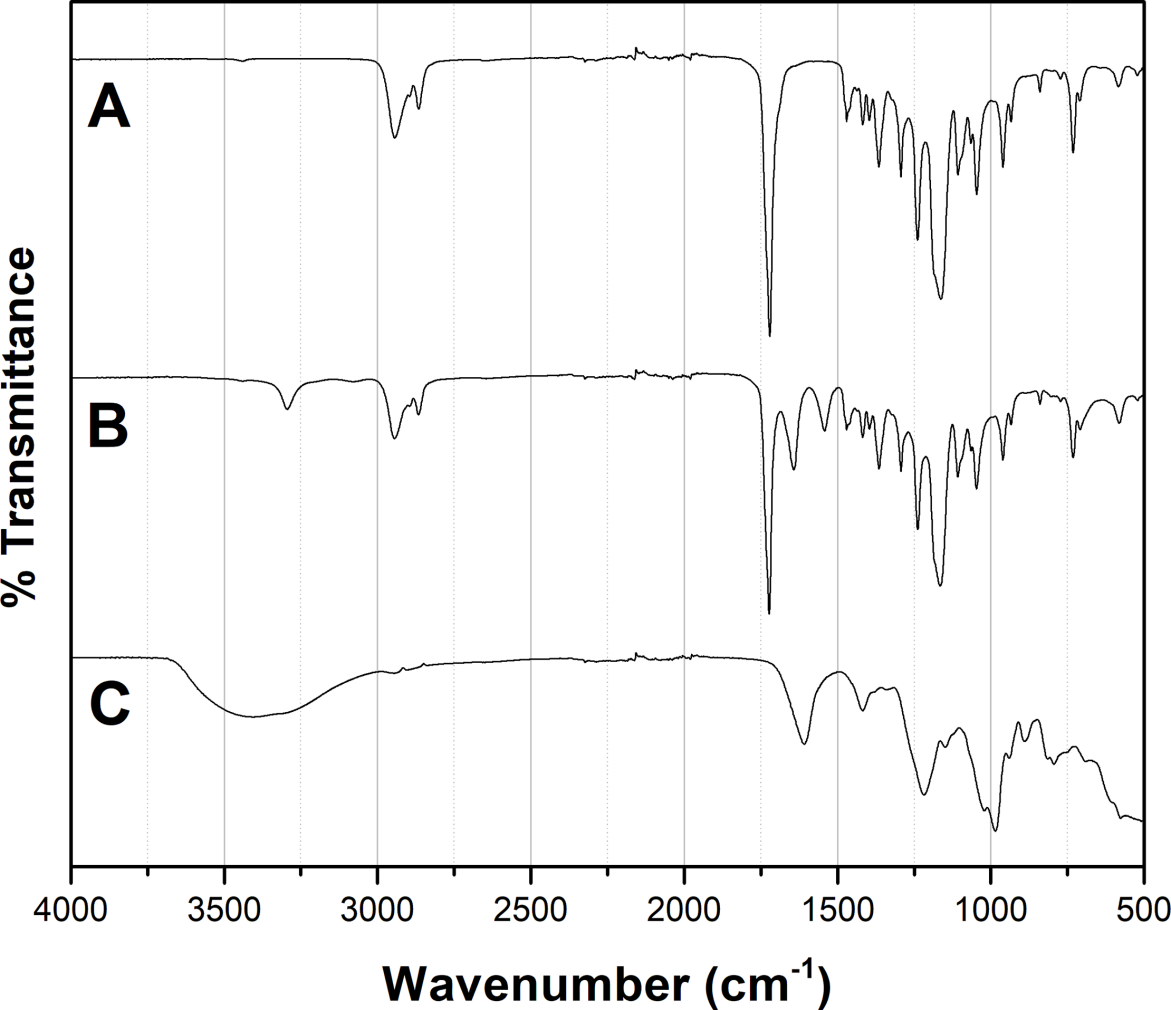
ATR-FTIR spectra of heparin and PCL mats. (A) PCL mat, (B) PCL-Hep-CL mat and (C) heparin powder. PCL-Hep-CL material presented a new peak at 1660 cm^-1^ (amide C = O stretching) and a band at 3400cm^-1^ (-OH stretching).

The ATR-FTIR spectra of PCL mat, PCL-Hep-CL mat and heparin powder are displayed in Fig 2. PCL and PCL-Hep-CL mats showed absorptions peaks at 2940 cm^-1^ and 2860 cm^-1^ representing -CH2- stretching. In addition, the absorbance of carbonyl group is shown at 1720 cm^-1^ for both mat types. The PCL-Hep-CL material presented a new peak at 1660 cm^-1^ and a band at 3400 cm^-1^ which correspond to an amide C = O and -OH stretching, respectively. These results suggest that heparin was successfully incorporated into PCL fibers by means of the crosslinking method.

#### 3.1.3 Distribution and surface immobilization of heparin in PCL mats

**Fig 3:**
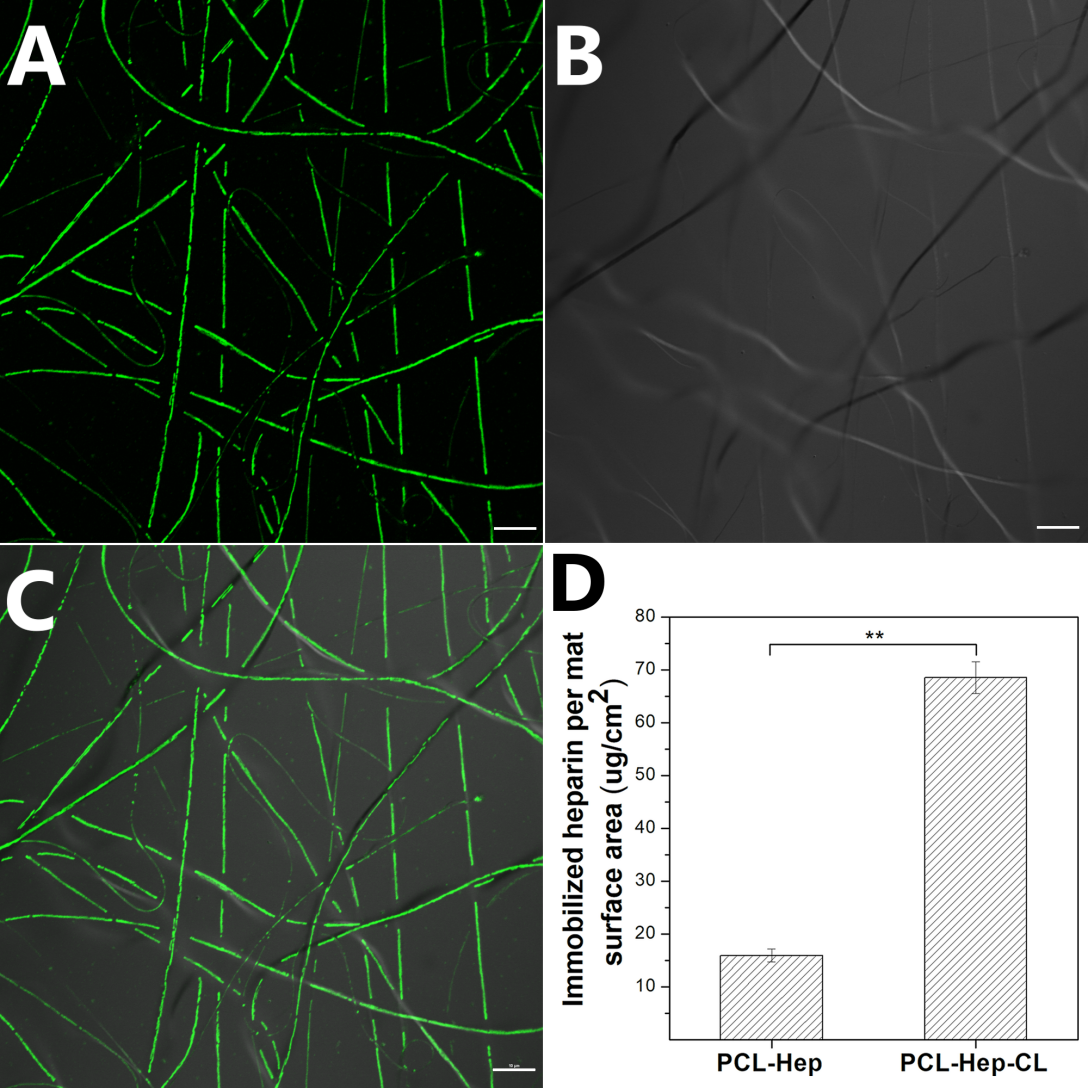
Heparin distribution and surface immobilization in PCL mats. Images represent the confocal (A), optical (B) and superposed (C) microscopy images of PCL-HepF mats obtained with an Olympus IX 81 Microscope. Scale bars are 10 μm for all images. (D) Immobilized amount of heparin present in PCL-Hep and PCL-Hep-CL mats per unit area corresponded to 15.92 μg ± 1.22, and 68.56 μg ± 3.02, respectively. Data represents means ± SE (n = 3). There were significant differences between the heparin present in the two types of mats (**, p<0.01).

Fig 3A and 3C show that the HepF conjugate is distributed along the entire length of PCL-HepF fibers. This also proves that HepF present in the DCM:MeOH solution is stable throughout the EHD process. Fig 3D shows the amount of immobilized heparin on PCL-Hep and PCL-Hep-CL per unit mat surface area, with values of 15.92 ± 1.22 μg/cm^2^, and 68.56 ± 3.02 μg/cm^2^, respectively. Taking into account the specimen’s weight, we estimate that 12.5 μg of heparin were immobilized in the PCL-Hep discs (geometrical area of 0.785 cm^2^). In addition, the covalent bonding of heparin to a PCL-Hep-CL mat of the same geometrical area and disc size led to more than a four-fold increase in the payload of heparin (53.82 μg). This increase in heparin content was expected to improve the binding affinity with HPV virus.

#### 3.1.4 Contact angle measurements

**Fig 4:**
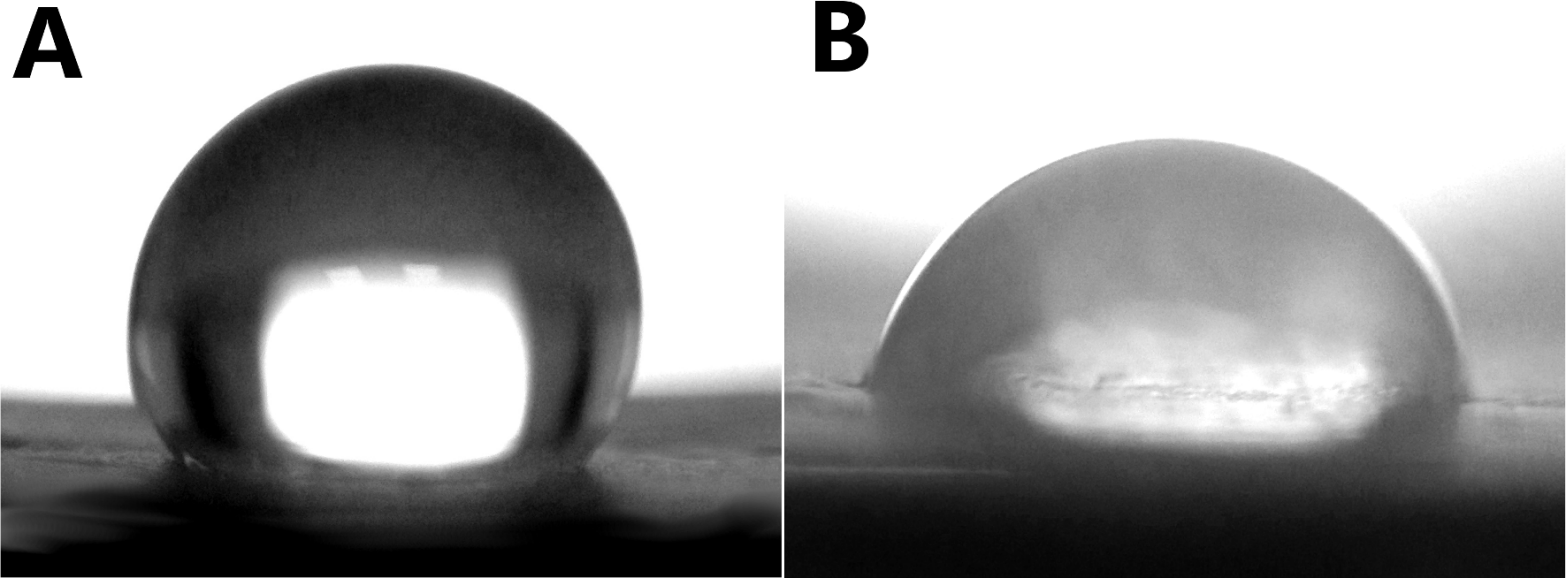
Contact angle images of fibrous materials. Representative images for contact angle measurements of PCL (A) and PCL-Hep (B) as collected mats, which values corresponded to 123 ± 0.8° and 92 ± 1.4°, respectively. Data represent means ± SD (n = 4).

In Fig 4, representative images of 5μL DI water droplets on the PCL (A) and PCL-Hep (B) mats are shown, which measured contact angles corresponded to 123 ± 0.8° and 92 ± 1.4°, respectively. Water droplets in PCL-Hep-CL were absorbed instantaneously once deposited onto them, thereby preventing the collection of any meaningful contact angle measurements. This is however, an indication of complete wettability i.e., a water contact angle of 0°.

#### 3.1.5 Thermal properties of PCL mats

**Fig 5:**
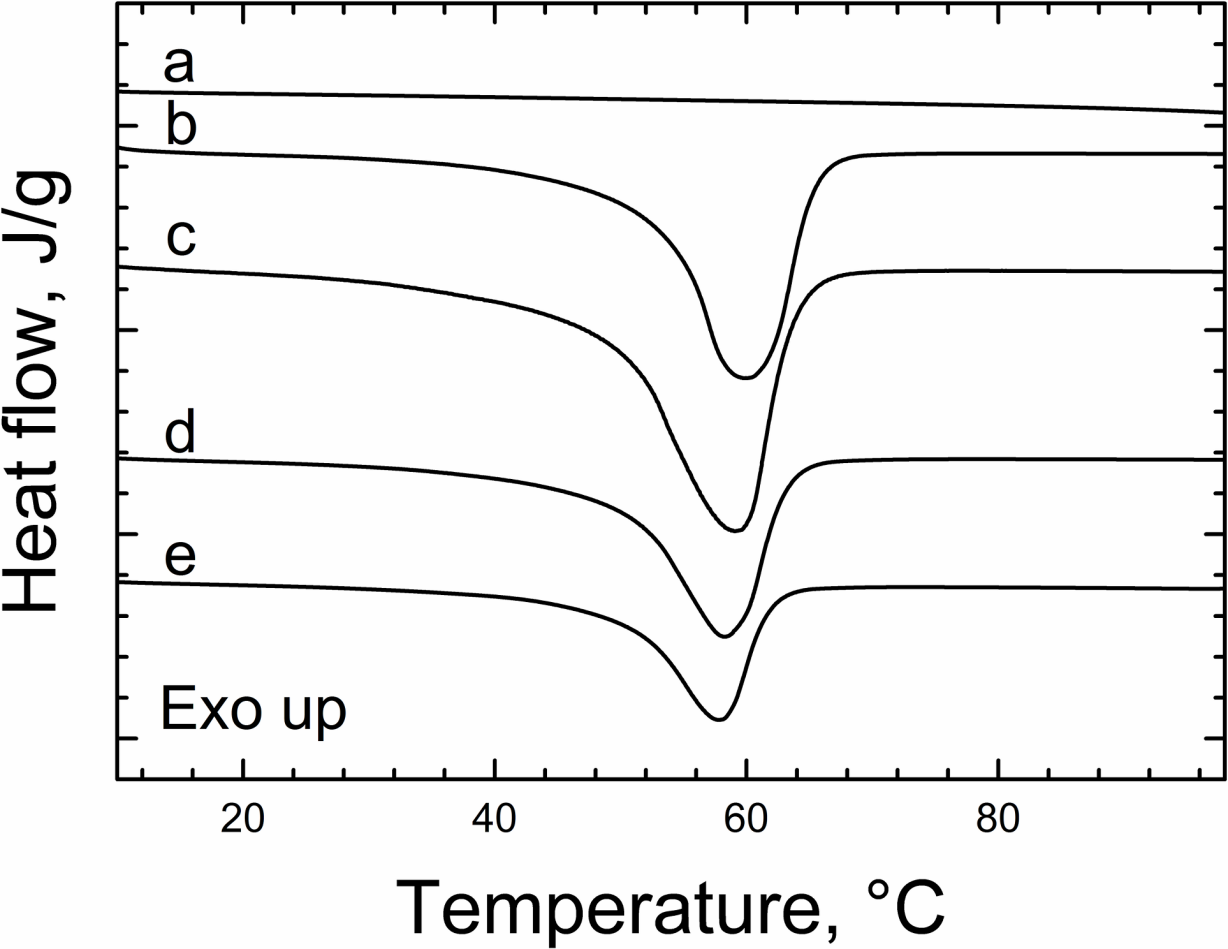
DSC thermograms of heparin and PCL samples. (a) Heparin powder, (b) PCL polymer pellets, (c) PCL-fibers, (d) PCL-Hep fibers and (e) PCL-Hep-CL fibers. All thermograms correspond to the second heating cycle.

DSC thermograms and thermal properties of the starting and the PCL fibrous materials are shown in Fig 5 and Table 1, respectively. The PCL fibers in this study were semi-crystalline, as confirmed by DSC analysis, showing a single melting temperature peak that corresponds to the melting point of PCL (~60°C). The T_m_ and X_c_ values for all the EHD-processed materials, presented lower values when compared to that of the as-received PCL pellets (Table 1), indicating that the EHD process led to lower degrees of polymer crystallinity.

**Table 1:** Thermal properties of PCL mats.

Sample	ΔH_m_ (J/g)	T_m_ (°C)	X_c_^a^ (%)
PCL polymer pellets	57.85	59.81	41.62
PCL fibers	51.08	59.21	36.75
PCL-Hep fibers	46.11	58.99	33.17
PCL-Hep-CL fibers	37.93	57.35	27.29

^a^ X_c_(%) = (ΔH_m_/ΔHm_o_)

ΔH_m_ = enthalpy of melting of sample

ΔHm_o_ = enthalpy of melting of fully crystalline PCL, 139 J/g.

#### 3.1.6 Cytotoxicity of fibrous mats

**Fig 6:**
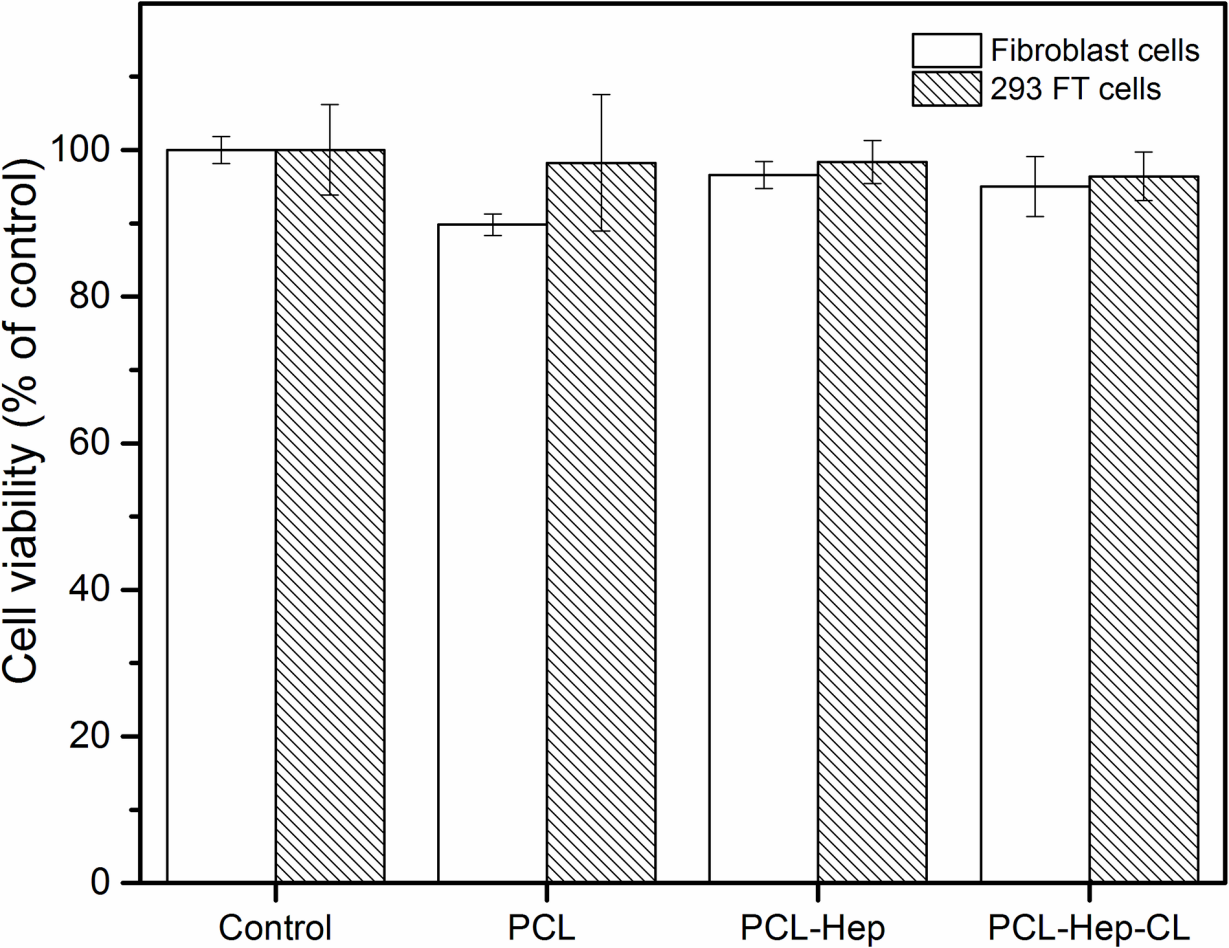
Cell viability of fibroblasts and 293FT cells on mats. Supernatants which were preincubated with PCL, PCL-Hep and PCL-Hep-CL mats were added to cells and incubated for 3 days at 37°C and 5% CO_2_. The WST-8 reagent solution was contacted with the samples for 1 hr prior to OD450 measurement with a BioTek Elx800 microplate reader. Cytotoxicity effects of the different materials were not statistically significant different when compared with their respective controls. Data represent means ± SE (n = 4).

Fig 6 shows the survival rate (%) of fibroblasts and 293FT cells after 3 days of incubation with PCL, PCL-Hep and PCL-Hep-CL supernatants. Significant cytotoxicity effects for both types of cells were not observed in all materials at 0.01 level when compared with the control (cells alone). In PCL-Hep-CL mats, heparin was covalently attached to the PCL fibers via the EDC/NHS coupling reaction. This method is broadly used for immobilization of bioactive molecules, due to its mild and non-cytotoxic reaction conditions [45]. Cytotoxicity results suggest that both the as-collected and the crosslinked PCL mats support cell viability and proliferation.

#### 3.1.7 Heparin stability on heparin-loaded mats

**Fig 7:**
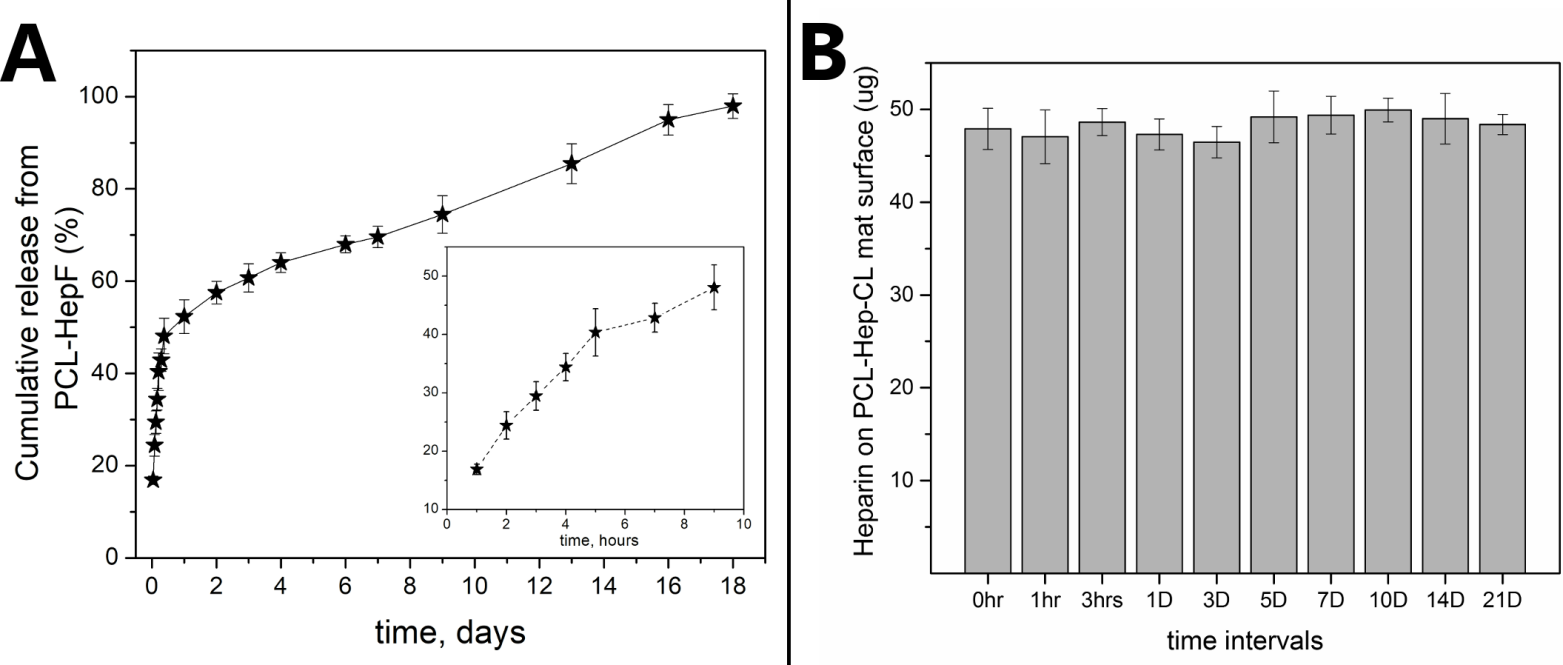
Heparin stability in heparin-loaded materials. (A) Cumulative release curves of heparin-fluorescent from PCL-HepF mats in PBS at 37°C. The inset shows the release profile during the first 9 hrs. Data represent means ± SD (n = 3). (B) Heparin content on PCL-Hep-CL mat surface after different incubation times in PBS at 37°C. Data represent means ± SE (n = 3).

Fig 7A shows the cumulative HepF percentage release over a period of 18 days. This study shows that 17 ± 1% of the heparin in the PCL-HepF mat was released in PBS during the first hour. In addition, 52 ± 4% was released during the first day of incubation, and almost all heparin loading was released by day 18. From the release profile, it is apparent that there is a “burst” release phase (Fig 7A, inset), followed by a delayed phase that lasts up to 430 hrs. Fig 7B shows the heparin content in PCL-Hep-CL as a function of incubation time. This figure shows that the amount of heparin initially present (~48 μg) was almost entirely preserved after an incubation period of 21 days, which is clearly indicative of the stability of immobilized heparin in the PCL-Hep-CL sample.

### 3.2 Heparin-loaded PCL materials interactions with HPV16

#### 3.2.1 Binding assays with GST-L1 protein

**Fig 8:**
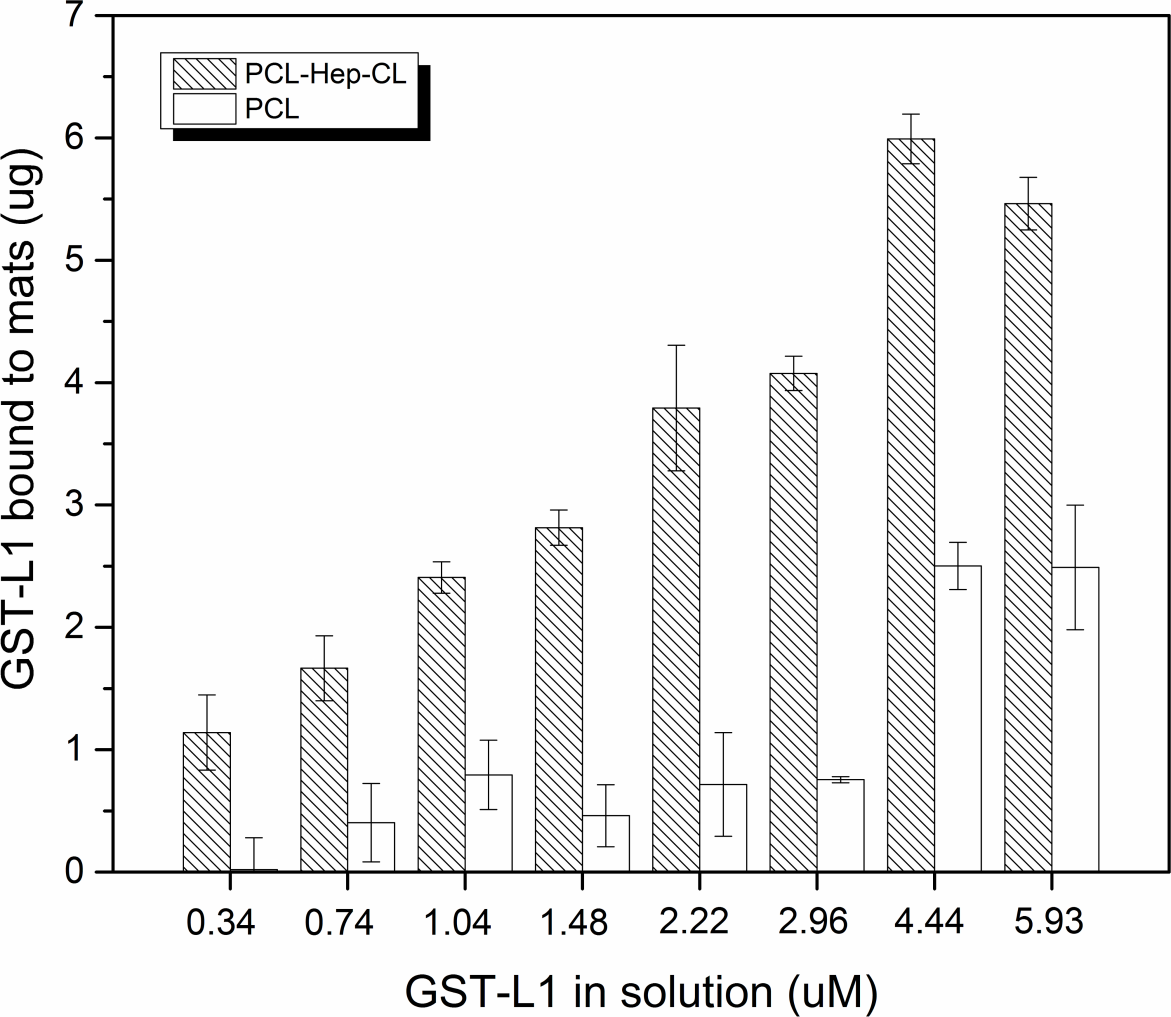
Binding of GST-L1 protein to PCL-Hep-CL mats. PCL-Hep-CL mat discs were incubated with increasing concentrations of GST-L1 protein. Western Blot was performed directly on the mats to quantify the amount of protein bound to the discs. Data represent means ± SE (n = 3).

The results of binding assays are shown in Fig 8. The amount of GST-L1 bound to PCL-Hep-CL mats increased with increasing GST-L1 concentration in solution. The bound protein amount reached a saturation plateau when the protein concentration was 4.4 μM. At this point, about 6 μg (74 picomoles) of protein per mat, remained bound. No further increase in binding was observable at higher GST-L1 concentrations. Considering the weight of the PCL-Hep-CL mat, the maximum amount of heparin bound per mat weight corresponded to a value equal to 2.4 mg/g. However, the same binding assay using PCL-Hep mats yielded no significant results. As shown in their release profile (Fig 7A), the direct quantification of L1 protein bound to PCL-Hep mats was masked by the extended release of heparin to the liquid medium. In spite of the fact that formation of the L1-heparin complex is *a priori* a possibility, it was not detected by means of the proposed quantitative approach.

**Fig 9:**
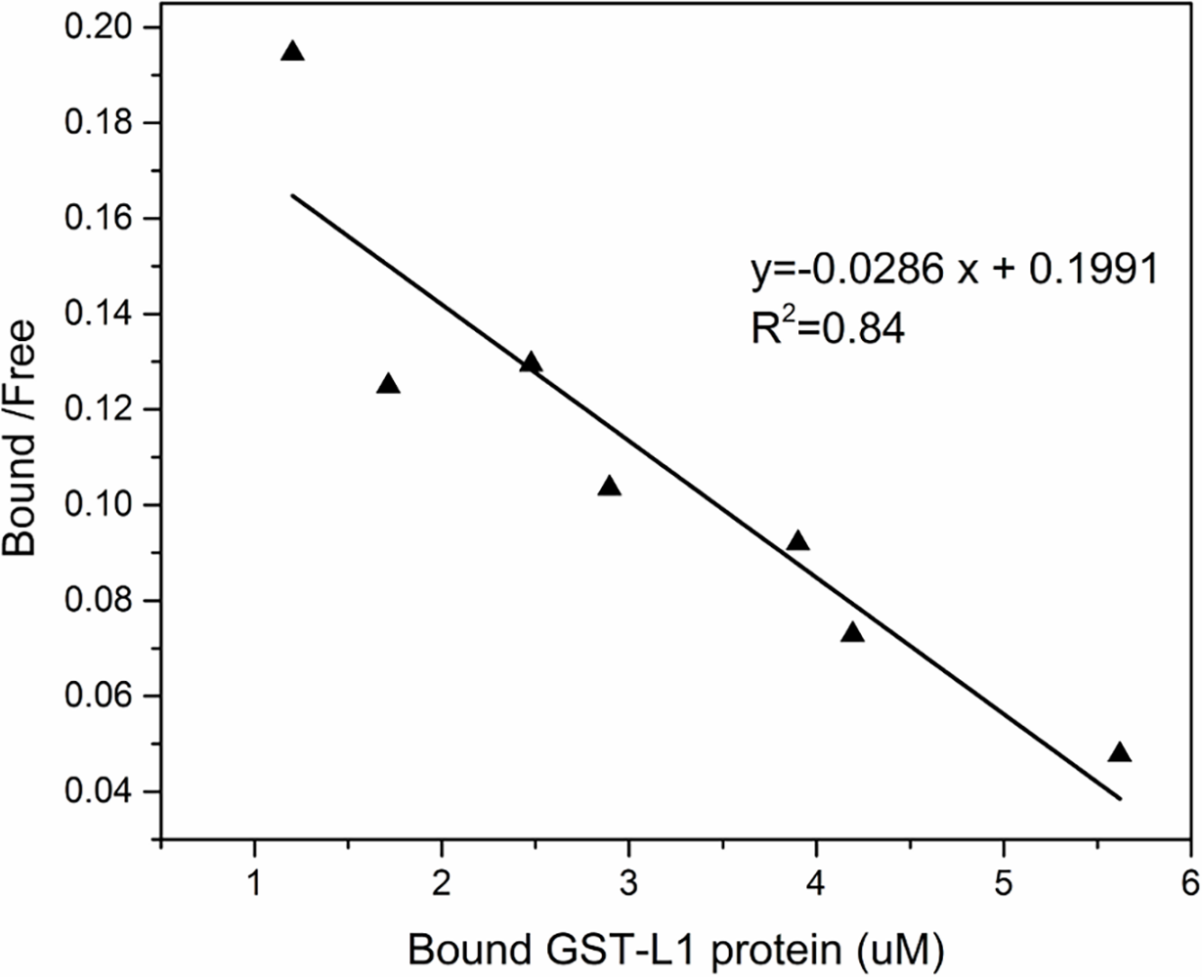
Scatchard plot for GST-L1 protein binding to PCL-Hep-CL mats. The amount of GST-L1 protein bound to PCL-Hep-CL mats was quantified by Western blot. The mean values of three experiments are plotted.

To further evaluate the affinity of GST-L1 proteins for the heparin present in PCL-Hep-CL mats, the dissociation constant (K_d_) was calculated via a Scatchard plot (Fig 9). Scatchard plots are generated by plotting the bound-to-free protein ratio versus the bound protein concentration. Bound protein concentration was calculated as the ratio of the amount of bound GST-L1 to sample volume. The mat discs used in these experiments had a geometrical surface area of 0.785 cm^2^, and a thickness of 150 μm. The slope of the straight line in the Scatchard plot is -1/K_d_, which corresponded to a K_d_ value of 35 μM.

#### 3.2.2 HPV16 PsV inhibition assay

**Fig 10:**
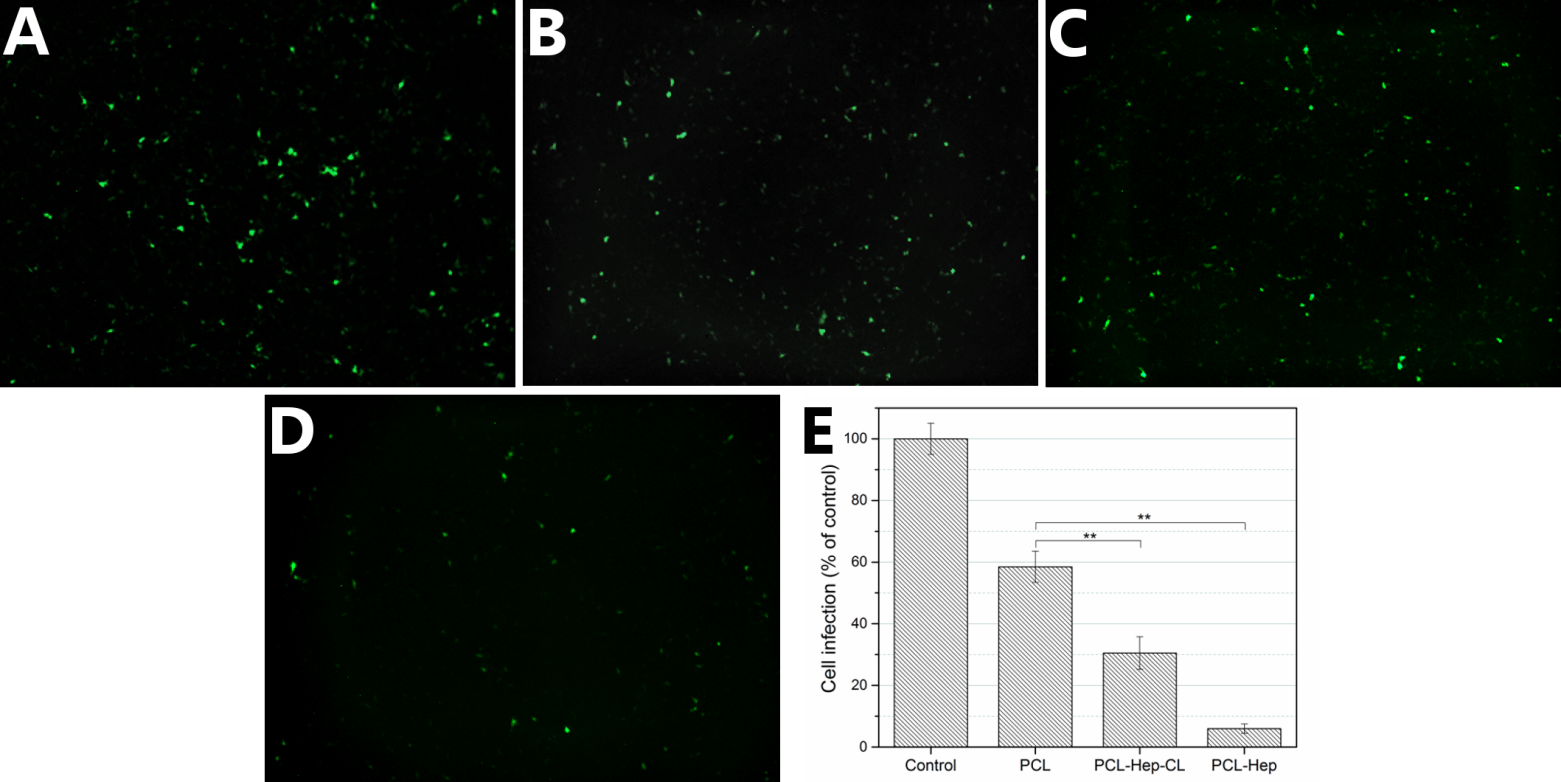
Infectivity assay with HPV16 PsVs and PCL mats. Confocal microscopy images of 293FT cells, transduced and expressing a GFP reporter plasmid corresponding to control (A), PCL (B), PCL-Hep-CL (C), and PCL-Hep mats (D). (E) Cell infection percentages of the different groups when compared to the control. Data represent means ± SD (n = 3). There were significant differences between the heparin loaded materials when compared with PCL (**, p<0.01).

Fig 10 shows the confocal images of 293FT infected cells expressing the GFP plasmid under each condition. Fig 10E shows the cell infection percentages of each group relative to the control, with corresponding values of 58.46 ± 5.05%, 30.50 ± 5.31%, and 6.01 ± 1.57% for PCL, PCL-Hep-CL, and PCL-Hep materials, respectively. Both of the heparin-loaded materials presented significant differences when compared to either the control or PCL mats. Interestingly, PCL-Hep-CL mats reduced cell infection by 70%; however, although the crosslinked materials possess a higher amount of heparin on its surface, it did not show an inhibitory effect as pronounced as for the case of the non-covalent material. The PCL-Hep material reduced cell infection with HPV16 PsVs by 94%, as shown by its lowest count of fluorescent cells.

## Discussion

The increase in fiber diameter in PCL-Hep mats is likely related to the change in the solution formulation before the electrospinning process. Methanol compared to DCM, has a higher boiling point temperature and lower surface tension. The increment of methanol affected the volatility and surface tension of the final solution, consequently increasing the fiber diameter of the electrospun product. Similarly, the surface roughness and diameter also increased for the case of PCL-Hep-CL fibers. This modification in PCL-Hep fibers after the EDC/NHS heparin crosslinking method was also evident in the work of other groups [46–49]. The increment in fiber diameter may be attributed to distention and swelling of the polymer caused by water intake. This phenomenon has been termed “crimp accentuation” in the textile industry [50], which also causes a decrease in the overall dimension of samples. After crosslinking, besides the increment in fiber diameters, the sample discs presented a 10% diameter decrease, and their weight increased by approximately a 26% when compared to their initial values before treatment. These effects have also been reported by other research groups when analyzing the impact of using crosslinking agents on EHD-processed collagen mats [48].

The surface of the heparin conjugated mats showed more hydrophilic characteristics than those created with PCL alone, which can be attributed to the highly hydrophilic sulfonic groups in heparin. The increased PCL-Hep-CL hydrophilicity was viewed as being highly desirable in this material. Ideally, a device acting as a virus capture should be as wettable as possible when in contact with biological fluids.

The lower crystallinity of EHD-processed fibers can be explained by the rapid solvent evaporation caused by the large drying surface area, which, in fact, prevents rearrangement of polymer chains [51,52]. Our results are also in agreement with the findings of other groups, in which the preparation of PCL fibers by EHD also showed a decrement in X_c_ values [53–55]. The X_c_ and T_m_ values were also reduced in PCL-Hep fibers (33.17% and 58.99°C, respectively). These changes in the thermal properties of PCL-Hep fibers seems to be due to the presence of heparin. Heparin (an amorphous linear polymer) showed the absence of a melting peak that is characteristic of crystalline phases (Fig 5A). Finally, it was found that the effect of heparin crosslinking in PCL-Hep-CL fibers resulted in an additional decrease in X_c_ (27.29%) and consequently, in a lowered T_m_ (57.35°C). Crosslinked polymer systems tend to be stable mechanically and thermally: once formed, they are difficult to break. This type of chemical modification introduces new moieties that can negatively impact the migration and diffusion of otherwise mobile polymer chains to the surface of the growing polymer crystal. The molecules in PCL-Hep-CL fibers thus have less mobility to rearrange and effectively crystallize, evidenced by its lowest X_c_ among all the PCL mats produced in this study. In addition, and even though the majority of crosslinked heparin is located at the surface of PCL-Hep-CL fibers, the massive changes in fibers’ morphology and dimensions caused by the crosslinking process is expected to be responsible for the additional decrement in thermal properties and polymer crystallinity.

The heparin release mechanism from PCL-Hep mats was investigated by measuring the exponent (*n*) in the general solute release equation proposed by Ritger and Peppas. For the case of cylindrical samples, this equation predicts that the mechanism for solute release is controlled by diffusion when n = 0.45, and of a zero-order kinetics type when n = 0 [42]. The cumulative HepF release curve was thus fitted to an allometric power growth equation (*y* = *kt^n^*), casting an *n* value equal to 0.28. This number, which clearly falls within the two mechanistic extremes of the drug release process, suggests the occurrence of “anomalous” transport i.e., an overlap of drug diffusion and polymer swelling phenomena [56]. This finding further corroborates that heparin in PCL-Hep is mostly physically adsorbed on the fibers’ surface. The stability of covalently immobilized heparin in PCL-Hep-CL material was shown to be higher than that of physically adsorbed or ionically immobilized heparin in PCL-Hep mats, as demonstrated in Fig 7B. Similar results support the proposal that covalently bonded heparin is long-term stable [33,36,57].

The binding constant found for the PCL-Hep-CL material falls within the same order of magnitude as those reported for heparin-protein L1 peptides, and other heparin-peptide interactions [16,58–60]. *Sun et al*. studied the interaction of heparin with five synthetic peptides of HPV16, obtaining binding constants in the order of 10^5^ M^-1^. The ability of HPV11 virus like particles (VLPs) to interact with heparin was investigated by heparin affinity chromatography [17]. Results showed that approximately 93% of bound VLPs were eluted, pointing to the high affinity and strong interactions of HPV L1 protein with heparin, which possessed a similar molecular weight to the one utilized in this manuscript. Our results however, besides demonstrating a strong L1 protein-heparin binding interaction, also show that the HPV L1 protein strongly interacts with the heparin immobilized in an engineered material, namely heparin-loaded PCL fibers. To the best of our knowledge, this has not yet been reported. In addition, it points to a potential opportunity to develop materials that actually capture the virus (e.g. tampons, condoms), not just inactivate them.

Previous studies have shown that sulfated polysaccharides such as heparin, cellulose sulfate, and dextran sulfate, can block the HPV infection [13,17,61]. For instance, high-molecular weight heparin has been tested as possible material to inhibit PsV binding to HaCaT cells [17]. Results showed a dose-dependent effect, and an IC_50_ heparin concentration of 0.3 μM. In addition, infection studies using COS-7 cells and HPV16 PsVs, carrying a GFP reporter plasmid showed that heparin present at a concentration of 0.14 mg/mL (5.6 μM) was found to suppress HPV16 infectivity by 50% [13]. From our results, we can estimate that after the infectivity assay, approximately 8% of heparin is released from PCL-Hep mats. This value corresponds to a concentration of heparin in solution of 0.3 μM, which translates into the significant observed HPV16 infection reduction of 94%. While two virus inhibition mechanisms are possible with these materials, the fact that the PCL-Hep material showed a higher impact in reducing infectivity lends credence to the idea that heparin’s mechanism of virus inhibition is more efficient when performed in soluble phase rather than only an immobilized phase.

## Conclusions

Two different approaches to produce heparin-loaded poly-ε-caprolactone (PCL) fibrous materials were applied in this study. Both materials showed good cell viability properties, and the incorporation of heparin into the fibers improved the hydrophilicity of the materials, and decreased polymer crystallinity. Our first material design method led to a conventional heparin release profile, and when infectivity assays were conducted with it, a 94% cell infection reduction with HPV16 PsVs was observed. The second sample production approach constituted an effective method to immobilize heparin on PCL, and had a high binding affinity for HPV16 L1 capsid (K_d_ = 35 μM), in the same range as those generally observed in relevant heparin-peptide complexes. This material also reduced HPV cell infection by 70%. The two proposed materials design options provide clues, for the first time to the best of our knowledge, for eventually making an effective device to combat HPV infection, either by direct association of the virus with heparin in solution, or via selective capture of the virus in the fibrous mat. These studies also suggest that this approach may be effective against HPV as well as other heparin-binding viruses.

## Supporting information

S1 Fig Schematic diagram of heparin conjugation onto PCL mats by means of EDC/NHS coupling reaction. Heparin is incorporated to the PCL fibers via the formation of a stable amide bond.
(TIF)

S1 Text Chemical synthesis process of PCL-Hep-CL mats. (PDF)
