# Supplementary figures and images for: Preparation and characterization of functionalized heparin-loaded poly-Ɛ-caprolactone fibrous mats to prevent infection with human papillomaviruses
Source: PLoS One. 2018 Jul 2;13(7):e0199925. doi: 10.1371/journal.pone.0199925 (PMC6028096; doi:10.1371/journal.pone.0199925)

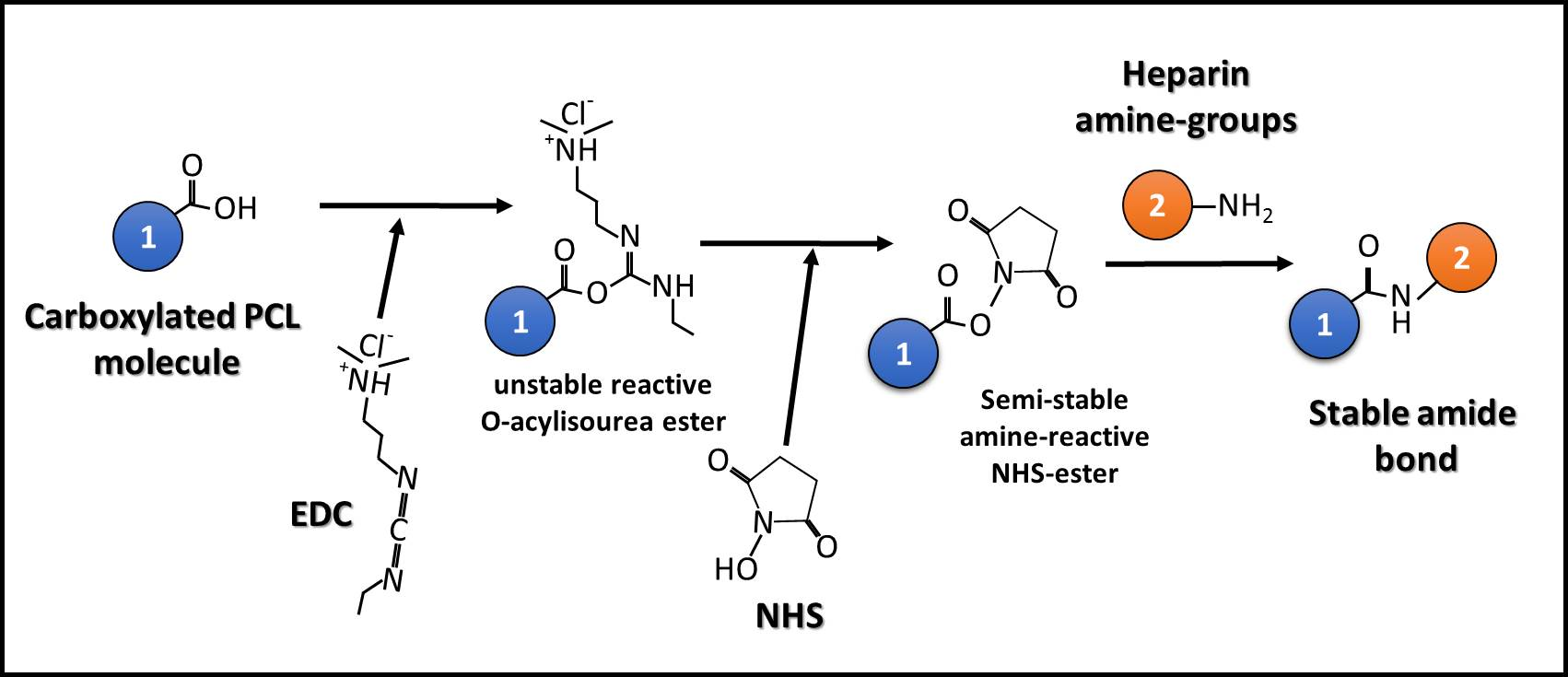

Supplement: S1 Fig — Heparin is incorporated to the PCL fibers via the formation of a stable amide bond. (TIF) [file pone.0199925.s001.tif]
