# Supplementary material for: Preparation and characterization of functionalized heparin-loaded poly-Ɛ-caprolactone fibrous mats to prevent infection with human papillomaviruses
Source: PLoS One. 2018 Jul 2;13(7):e0199925. doi: 10.1371/journal.pone.0199925 (PMC6028096; doi:10.1371/journal.pone.0199925)
Supplement: S1 Text — (PDF) [file pone.0199925.s002.pdf]

Chemical synthesis process of PCL-Hep-CL mats

The covalent immobilization of biomolecules on polymeric fibers often requires the activation of exposed functional groups on the surface, the biomolecules or both. Primary amines and carboxyl groups are frequently used as anchoring points for this type of reactions. The 1-ethyl-3-(3-dimethylaminopropyl) carbodiimide (EDC), a zerolength crosslinking agent, and N-hydroxysuccinimide (NHS) are among the most used reagents to activate polyester surfaces.

The EDC/NHS method is commonly used because of its mild and non-cytotoxic reaction conditions [1]. In general, this surface functionalization method consists of three steps: 1) pretreatment of the polyester mat to increase its hydrophilicity, i.e. by partial hydrolysis, 2) activation of the polyester chain with EDC/NHS intermediates, and 3) binding of the polyester surface to the biomolecule. The surface immobilization of heparin constitutes a well-known example of covalent bonding via the EDC/NHS coupling reaction [2–5]. Wang *et al.* functionalized EHD-processed fibrous PCL scaffolds with heparin to investigate their use as potential vascular grafts [6]. S1 Fig shows a schematic representation of heparin conjugation onto EHD-processed PCL fibers by means of the EDC/NHS method. The presence of NHS, besides increasing the overall reaction efficiency, converts an unstable amine-reactive EDC-formed intermediate, into a semi stable amine-reactive NHS ester [7,8]. The result is that heparin becomes attached to PCL fibers via formation

of a stable amide bond between PCL carboxyl groups and amine groups present in heparin.

References

1. Olde Damink LH, Dijkstra PJ, van Luyn MJ, van Wachem PB, Nieuwenhuis P, Feijen J. Cross-linking of dermal sheep collagen using a water-soluble carbodiimide. Biomaterials. 1996;17: 765–73. doi:10.1016/0142-9612(96)81413-X
2. Gümüdereliolu M, Aday S. Heparin-functionalized chitosan scaffolds for bone tissue engineering. Carbohydr Res. 2011;346: 606–613. doi:10.1016/j.carres.2010.12.007
3. Liao D, Wang X, Lin PH, Yao, Qizhi CC. Covalent linkage of heparin provides a stable anti-coagulation surface of decellularized porcine arteries. J Cell Mol Med. 2009;13: 2736–2743. doi:doi:10.1111/j.1582-4934.2008.00589.x.
4. Lee J, Yoo JJ, Atala A, Lee SJ. Controlled heparin conjugation on electrospun poly(-caprolactone)/gelatin fibers for morphology-dependent protein delivery and enhanced cellular affinity. Acta Biomater. Acta Materialia Inc.; 2012;8: 2549–2558. doi:10.1016/j.actbio.2012.03.030
5. Ye L, Wu X, Mu Q, Chen B, Duan Y, Geng X, et al. Heparin-Conjugated PCL Scaffolds Fabricated by Electrospinning and Loaded with Fibroblast Growth Factor 2. J Biomater Sci Polym Ed. 2011;22: 389–406. doi:10.1163/092050610X487710

6. Wang Z, Sun B, Zhang M, Ou L, Che Y, Zhang J, et al. Functionalization of electrospun poly(Ɛ-caprolactone) scaffold with heparin and vascular endothelial growth factors for potential application as vascular grafts. J Bioact Compat Polym. 2012;28: 154–166. doi:10.1177/0883911512469707
7. Casper CL, Yang W, Farach-Carson MC, Rabolt JF. Coating electrospun collagen and gelatin fibers with perlecan domain I for increased growth factor binding. Biomacromolecules. 2007;8: 1116–1123. doi:10.1021/bm061003s
8. Thermo. Thermo Scientific Pierce Crosslinking Technical Handbook. Ebooks. 2009.
